# Exploring Cancer‐Associated Fibroblasts in OSCC and OPMDs: Microenvironment Insights. Scoping Review

**DOI:** 10.1111/odi.15275

**Published:** 2025-02-10

**Authors:** Samuele Sutera, Olga Anna Furchì, Monica Pentenero

**Affiliations:** ^1^ Oral Medicine and Oral Oncology Unit, Department of Oncology University of Turin Turin Italy

**Keywords:** cancer associated fibroblasts, carcinogenesis, malignant transformation, oral potentially malignant disorder, oral squamous cell carcinoma, tumor microenvironment

## Abstract

**Introduction:**

The tumor microenvironment (TME) plays a crucial role in oral squamous cell carcinoma (OSCC) and oral potentially malignant disorders (OPMDs). Despite progress, the mechanisms behind the TME‐epithelial cell interaction remain unclear. Cancer‐associated fibroblasts (CAFs), the most abundant cells in the TME, require further study.

**Material and Methods:**

We conducted a scoping review, searching for clinical and vivo studies that discuss the role of CAFs in OSCC and OPMDs progression.

**Results:**

From 1152 PubMed results, 29 studies met the inclusion criteria. CAFs, identified as αSMA+ cells, interact with the TME and epithelial cells by secreting various molecules. In OSCC, CAF signals contribute to a pro‐tumorigenic environment, and CAF numbers positively correlate with tumor grade, size, stage, aggressiveness, and mortality. While limited data exist on CAFs in OPMDs, they seem linked to malignant transformation risk.

**Discussion:**

CAFs are critical in OSCC pathophysiology, but the complex intercellular mechanisms are not fully understood. Currently, CAFs are not part of clinical decision‐making, but emerging evidence suggests they could represent a promising new approach in managing OSCC and OPMDs.

**Conclusion:**

Future research should aim to gain a deeper understanding of how CAFs contribute to OSCC progression and their role in OPMDs pathophysiology.

## 1 Introduction

Oral squamous cell carcinoma (OSCC) is a common epithelial tumor, yet it has a disappointing 5‐year survival rate (Fang et al. 2017).

According to the World Cancer Research Fund International (2020 data), OSCC ranks as the 16th most common cancer overall, representing about 4% of all malignancies. It is the 11th most common in men and the 18th in women, with the World Health Organization (WHO) estimating 300,000–400,000 new cases annually worldwide, resulting in approximately 150,000 deaths (Bagordakis et al. 2016; Haque et al. 2019).

The 5‐year survival rate is about 50% and has not improved much in decades, despite advances in research and treatments. Notably, survival depends on tumor stage: stages I and II have around a 90% rate, while stages III and IV drop to 40% due to recurrence and metastasis (Sim, Hwang, and Ahn 2019; Li et al. 2019; Boxberg et al. 2019).

Tobacco, alcohol, or a combination of both are major risk factors for OSCC, causing about 90% of cases. Additionally, oral potentially malignant disorders (OPMDs) also significantly increase OSCC risk. Common OPMDs include oral leukoplakia (OL), oral lichen planus (OLP), graft versus host disease (GVHD), proliferative verrucous leukoplakia (PVL), systemic/discoid lupus erythematosus (SLE/DLE), and actinic cheilitis (AC). Oral epithelial dysplasia (OED) is not considered a separate entity but rather a step in the carcinogenesis process (Warnakulasuriya et al. 2021).

Proper follow‐up of patients with OPMDs is crucial for early OSCC diagnosis and improving survival odds. Paradoxically, widely accepted criteria for the diagnosis and management of OPMDs are still lacking, which can lead to patient management variations depending on the clinician's background (Pentenero et al. 2021, 2022).

Many studies have sought to identify prognostic factors, like biomarkers, to guide OSCC treatment or predict OPMD transformation, but no universally accepted criteria exist yet.

Research initially centered on cancer cells, but recently, focus has shifted to the tumor microenvironment (TME) and its interaction with cancer cells. However, few studies have explored the ME of OPMDs (Li et al. 2015; Vered et al. 2019; Sun et al. 2019; Deepthi et al. 2019; Chang and Lee 2024; Steffen et al. 2024).

The mutual crosstalk between the TME and cancer cells is recognized as critically important in carcinogenesis, development, and cancer progression, including OSCC. However, the regulatory mechanisms involved still require full elucidation (Bello et al. 2011; Bagordakis et al. 2016; Kartha et al. 2016; Chawla, Urs, and Augustine 2017; Takahashi et al. 2017; Okuyama et al. 2018; Gu et al. 2019).

The TME comprises various cell types, including fibroblasts, immune cells, pericytes, and endothelial cells, each with a specific influence on cancer. Among these, cancer‐associated fibroblasts (CAFs) are the most abundant stromal cells in the TME (Kayamori et al. 2016).

To date, the biological effects of CAFs in OSCC progression and metastasis are generally acknowledged. CAFs interact with tumor cells by secreting numerous molecules, many of which have been studied. However, there is a lack of knowledge regarding the underlying mechanisms and signaling pathways by which CAFs influence OSCC prognosis. The literature agrees that further studies on CAFs and their activity in the TME are needed (Chandralekha Selvakumar, Auxzilia Preethi, and Sekar 2022; Sakai et al. 2022; Wang et al. 2023).

We conducted a scoping review (Lau and Kuziemsky 2017) to summarize the current knowledge on the role of CAFs in the TME of OSCC and OPMDs, focusing on their influence from carcinogenesis to tumor dissemination.

## 2 Materials and Methods

### 2.1 Study Design

A scoping review was conducted following, where applicable, the guidelines of the PRISMA‐ScR Checklist (Page et al. 2021). The goal was to assess the role of CAFs in the TME of OSCC and OPMDs. The review aimed to identify and summarize existing literature on this topic.

### 2.2 Eligibility Criteria

We included original in vivo studies that investigated CAFs in the context of OSCC and OPMDs. Only articles published in English and available as full texts were considered. We excluded reviews, studies focusing solely on the general microenvironment without specific analysis of CAFs, studies not related to OSCC or OPMDs, or those where data about the oral cavity could not be separated from other anatomical regions (such as the pharynx). Additionally, in vitro research and studies based on animal models were excluded (Table 1).

**TABLE 1:** Inclusion and exclusion criteria.

	Inclusion criteria	Exclusion criteria
Study design	Original research and clinical observational studies	Case reports, editorials, letters, and reviews
Population	In vivo research focusing on OSCC or OPMDs	In vitro research, animal model research, and studies that include pharyngeal diseases where data cannot be separated from the oral cavity
Language	Publications in English	Publications not in English or not available in full text
Relevance	Studies that directly address the role of CAFs in the context of OSCC or OPMDs	Studies that do not provide specific data on CAFs or do not meet the research question's criteria

### 2.3 Search Strategy

A comprehensive search was conducted in PubMed using the query terms: “Tumor Microenvironment AND (mouth neoplasms OR oral lichen OR leukoplakia OR oral lichenoid OR dysplasia OR GVHD OR lupus).” The search was limited to studies published up until May 2024.

### 2.4 Study Selection

Two review Authors (SS and MP) independently screened the titles and abstracts for relevance. Full texts of potentially relevant studies were then reviewed to determine eligibility. Any disagreements were resolved by discussion.

### 2.5 Data Extraction

In each paper, we collected data on CAF markers, mediators of intercellular crosstalk, clinical‐pathological endpoints related to OSCC, and the role of CAFs in OPMDs (when available). The data were then synthesized to provide a comprehensive overview of the role of CAFs in OSCC and OPMDs.

### 2.6 Quality Assessment

The quality of the included studies was assessed using criteria based on study design, sample size, methods, and reporting. However, due to the heterogeneity of the studies, a formal meta‐analysis was not feasible.

## 3 Results

### 3.1 Study Selection

**FIGURE 1:**
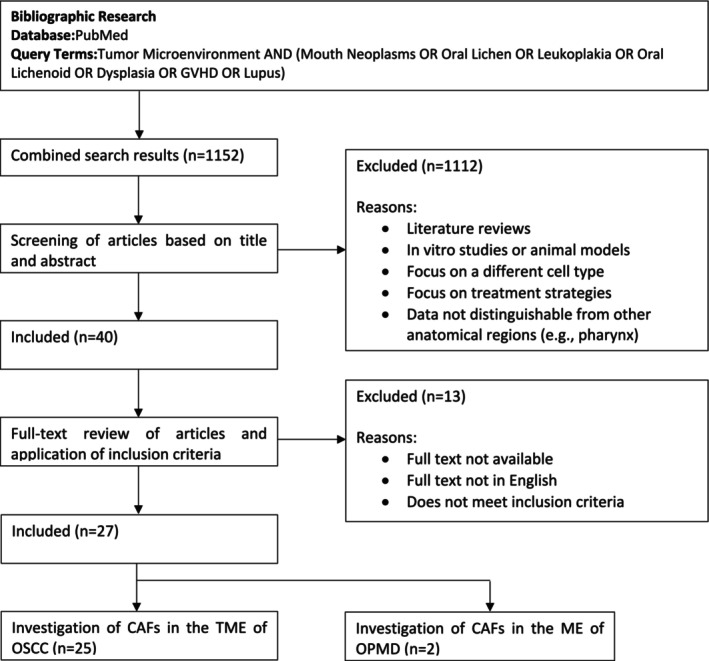
Flow diagram for paper selection process.

The initial search yielded 1152 records. After screening titles and abstracts for relevance, 40 papers were assessed for eligibility. Of these, 27 studies met the inclusion criteria after full‐text evaluation and were included in the review (Figure 1).

### 3.2 Study Characteristics

The included studies varied in design, encompassing both in vivo and clinical observational studies. Twenty five studies focused on OSCC, while two investigated the role of CAFs in OPMDs. Most of the studies were published in recent years, reflecting the growing interest in this research area.

### 3.3 Biological Information

Myofibroblasts are differentiated contractile cells that are normally found in the subepithelial layer of mucosal tissue. They secrete extracellular matrix components, cytokines, proteases, and proangiogenic factors. In the tumor stroma, there is a subpopulation of cells with a myofibroblast‐like phenotype known as CAFs (Dourado, Porto, et al. 2018).

CAFs are phenotypically similar to myofibroblasts. However, whereas myofibroblasts play a central role in wound healing and embryogenesis under physiological conditions, CAFs persist in pathological conditions. They establish a close relationship with carcinoma cells and contribute to tumor progression (Vered et al. 2019).

As mentioned, CAFs are the most abundant stromal cells in the TME and represent a heterogeneous population of irreversibly activated fibroblasts. CAFs are primarily derived from normal fibroblasts (NFs) and are activated by cancer cell‐derived cytokines. However, other cell types can also acquire fibroblast markers during carcinogenesis, including bone marrow‐derived mesenchymal stem cells, circulating fibrocytes, tissue adipocytes, and endothelial cells (Ding et al. 2018). CAFs are distinguishable from other cells by their spindle‐shaped morphology and the expression of α‐smooth muscle actin (α‐SMA), which is the most commonly used marker for detecting CAFs. Detailed analysis of the CAF proteome compared to NFs from adjacent tissue revealed higher intracellular levels of α‐SMA and an elongated mesenchymal morphology (Principe et al. 2018).

Since not all CAFs express specific markers (e.g., α‐SMA) to the same extent, it is suggested that CAFs with different marker expression patterns may play distinct roles within the TME (Zhao et al. 2020).

Summarizing the current knowledge, a high presence of CAFs is associated with adverse prognostic factors, including advanced disease stage, tumor grade, depth of invasion, and vascular, lymphatic, and neural invasion. Additionally, it correlates with extra‐nodal metastatic spread, recurrence, shorter time to disease relapse, and overall decreased survival. CAFs exert their protumorigenic effects by secreting a broad array of molecules that can directly influence cancer cell behavior, stimulating proliferation, invasion, and metastasis (Takahashi et al. 2017; Dourado, de Oliveira, et al. 2018; Wang et al. 2023).

Consequently, CAFs have been supposed to be a reliable marker for assessing and prognosticating OSCC. The relationship between CAF concentration and OSCC prognosis has been extensively studied, with some authors suggesting that CAF concentration is an independent prognostic factor (Ding et al. 2014; Li et al. 2015; Takahashi et al. 2017).

Given the recognized importance of CAFs in the TME of OSCC, many studies have investigated this cell type from various perspectives, including histological features, distribution, the relationship between CAF density and clinical outcomes, and the previous presence of OPMDs, among other factors. Despite the significant advances in understanding the origin and role of CAFs in the TME and their interactions with cancer cells, characterizing their heterogeneity and fully comprehending their mechanisms remains incomplete (Zhao et al. 2020).

### 3.4 Markers

Given the wide heterogeneity of potential CAF precursors and the variety of markers used to characterize CAFs, several authors have explored which marker or combination of markers best identifies this cell type. The primary markers considered include α‐SMA, fibronectin, FSP1, HHF35, vimentin, podoplanin, and PDGFRβ. The co‐expression of several markers (CAF immunophenotype) is more common in well‐differentiated tumors, although no significant associations with clinicopathologic parameters have been reported.

The role of PDGFRβ in OSCC is particularly noteworthy. It is highly expressed in numerous fibroblasts within the stroma surrounding tumor islands in most OSCC cases, whereas no PDGFRβ expression has been detected in tumor cells. This molecule is also known to be relevant in other cancers, including adrenocortical carcinoma, bladder urothelial carcinoma, colon adenocarcinoma, kidney renal clear cell carcinoma, liver hepatocellular carcinoma, lung adenocarcinoma, lung squamous cell carcinoma, pancreatic adenocarcinoma, and prostate adenocarcinoma. Some authors suggest that PDGFRβ is a consistent marker of stromal activation throughout both early and late stages of OSCC progression and could be valuable for isolating and further characterizing stromal fibroblasts in OSCC (Kartha et al. 2016; Dourado, Porto, et al. 2018).

The co‐expression of different markers, such as CD86 and α‐SMA, has been associated with CAF‐rich tumors and recognized as a negative prognostic factor for survival (*p* < 0.05) (Vered et al. 2019).

To date, the most commonly used marker in studies to identify CAFs is α‐SMA.

### 3.5 Mediators

Since CAFs influence cancer through molecules and extracellular vesicles (EVs) secreted into the extracellular matrix, comparing the secretomes of CAFs and NFs may reveal how CAFs promote a more aggressive cancer phenotype. Several potential mediators have been identified, including fibronectin type III domain‐containing 1 (FNDC1), serpin peptidase inhibitor type 1 (SERPINE1), stanniocalcin 2 (STC2), and lysyl oxidase (LOX), which are consistently upregulated in CAFs. In contrast, elastin (ELN), matrix metallopeptidase 3 (MMP3), and xylosyltransferase 1 (XYLT1) are overexpressed but not to a statistically significant extent. These proteins are primarily involved in extracellular matrix organization and disassembly, as well as collagen metabolism.

In this section, we list the main molecules reported in the literature and summarize the current knowledge about their roles in the TME, particularly in the interactions between CAFs and OSCC.

Firstly, increased expression of type I collagen N‐terminal propeptide (PINP) in both the overall stroma and the invasive front is associated with significantly shorter disease‐free survival compared to patients with lower expression (Bagordakis et al. 2016; Liu et al. 2023).

Rho‐associated coiled‐coil kinase 2 (ROCK2), an oncoprotein involved in cytoskeleton regulation, has been studied as a prognostic marker in solid tumors, including OSCC. It is expressed in both CAFs and cancer cells, with CAF overexpression suggesting its importance in the TME. High ROCK2 levels are linked to male gender, alcohol consumption, advanced stage, and high CAF density in the stroma, but not to smoking, tumor site, treatment, histological grade, margin status, or recurrence. Though not an independent prognostic factor, high ROCK2 expression is associated with shorter disease‐specific survival (DSS) and worse prognosis, especially with high CAF concentration (Dourado, de Oliveira, et al. 2018).

Non‐coding RNAs (ncRNAs) are a class of molecules recently studied for their impact on the TME. These single‐stranded RNAs, which don't code for proteins, are divided by their length into microRNAs (miRNAs, under 200 nucleotides) and long ncRNAs (lncRNAs, over 200 nucleotides). While ncRNAs are known to be crucial for physiological cellular processes, they are increasingly linked to pathological processes, including cancer pathogenesis and disease progression. Although research on ncRNAs in tumor stroma cells is limited and the function of ncRNAs in CAFs remains unclear, several lncRNAs are known to be upregulated, while others are downregulated in OSCC. To understand the lncRNA signature in stromal fibroblasts, lncRNA profiles in NFs and CAFs have been compared. LncRNAs like LOC400221 and SLC16A1‐AS1 are notably upregulated in OSCC, influencing the TME by increasing markers such as α‐SMA, vimentin, and N‐cadherin, and activating CAFs. This activation promotes tumor growth and worsens prognosis and survival. LncRNA FTX is tied to cancer proliferation and abnormal metabolism. Some lncRNAs are being considered for diagnostic and therapeutic use in OSCC (Ding et al. 2018; Feng et al. 2020; Li et al. 2023). MiRNAs, abundant in exosomes due to their small size, interact with OSCC cells and influence their behavior, as regulated by CAFs in the TME. Various miRNAs have either carcinogenic or carcinostatic effects. For example, miR‐146b‐5p can exhibit both pro‐ and anti‐carcinogenic activity depending on the cancer type, specifically, it is linked to promoting a malignant phenotype in OSCC (He et al. 2023). Of particular interest in OSCC is miR‐382‐5p, one of the most upregulated miRNAs in CAFs compared to normal fibroblasts. Its overexpression is associated with increased OSCC cell migration and invasion (Sun et al. 2019).

NOTCH signaling controls cell proliferation, apoptosis, and differentiation through four receptors (NOTCH1‐4). Dysregulation of this pathway is linked to various cancers, including OSCC. Different receptors have distinct roles; for example, NOTCH1 acts as a tumor suppressor, while NOTCH3 promotes tumor growth. In OSCC, NOTCH3 is found in about one‐third of CAFs, especially near the tumor's edge, but is rarely expressed in cancer cells. NOTCH3+ CAFs are associated with advanced disease, larger tumors, lymph node metastasis, and poorer prognosis. This may be due to NOTCH3+ CAFs enhancing vessel sprouting as NOTCH3 expression in CAFs significantly correlates with micro‐vessel density in the cancer stroma. Angiogenesis is a critical factor in cancer proliferation and metastasis. Additionally, cancer cells can also induce NOTCH3 expression in neighboring CAFs through cell‐to‐cell contact, boosting angiogenesis and tumor growth (Kayamori et al. 2016).

In summary, the interactions between CAFs and OSCC cells in the TME are mediated by a complex array of molecules, including proteins, ncRNAs, and signaling pathways. Understanding these molecular players not only sheds light on the mechanisms driving tumor progression but also could highlight potential therapeutic targets for improving patient outcomes in OSCC.

### 3.6 Relation With Other Cell Types

As previously mentioned, CAFs engage in complex cross‐talk with surrounding cells and are known to shape a pro‐tumoral microenvironment that promotes tumor progression. CAFs interact directly with cancer cells, as well as with other cells within the TME, including immune cells and vascular endothelial cells (Takahashi et al. 2017).

Many forms of intercellular communication have been identified, with potentially more to be discovered in the future. Among these, the exosome delivery system is particularly significant. CAF‐derived exosomes transport signaling molecules to adjacent cells, including growth factors, hormones, and cytokines. These delivered molecules can regulate gene expression in tumor cells, promote epithelial‐mesenchymal transition (EMT), and contribute to malignant transformation, tumor proliferation, invasion, and metastasis. Dysregulation of miRNAs in CAFs is thought to influence the content of these exosomes. Additionally, modifications in the microenvironment can lead to drug resistance (Okuyama et al. 2018; Sun et al. 2019; Zhao et al. 2023).

Analyzing the relationship between CAFs and inflammatory infiltrates reveals that CAF concentration is inversely correlated with the overall density of the inflammatory infiltrate. Conversely, CAFs are positively correlated with the cumulative density of regulatory T cells (Tregs) (Foxp3+), M2 tumor‐associated macrophages (TAMs) (CD163+), and potential Treg‐inducing immune cells (CD80+). In other words, the data highlight the association between CAFs and inflammatory components that are known to promote tumorigenesis. This evidence underscores that CAFs are linked to poorer clinical outcomes, including increased recurrence and reduced survival (Bello et al. 2011; Dayan et al. 2012).

A close relationship has been described between CAFs and M2 TAMs. CAFs contribute to the recruitment, proliferation, and M2 polarization of macrophages within the TME. They accomplish this through the increased expression of IL‐6 and CXCL8, which support M2 macrophage polarization, and TGF‐β, which facilitates the recruitment and retention of macrophages in the TME and enables effective tumor evasion of the host immune system. The ratio of CAFs to M2 TAMs initially increases and then decreases, following a curve. As CAFs transition from absent to sparse and then to a concentrated presence in the TME, the number of TAMs gradually increases. However, when CAFs become extensively and continuously concentrated, the density of TAMs decreases. This observation suggests that an excessive imbalance in TME components, such as a high concentration of CAFs, may induce distributional and/or functional changes in other components, including TAMs (Takahashi et al. 2017; Yang et al. 2022).

### 3.7 Clinical‐Pathological Endpoints

#### 3.7.1 Age and Gender

The data do not support a statistically significant relationship between CAF concentration and age or gender (Li et al. 2015; Sun et al. 2019).

#### 3.7.2 Tumor Grade

Based on the available studies, α‐SMA expression (indicating CAF concentration) and the microscopic grade of the tumor appear to be statistically significantly correlated (Akrish et al. 2017; Takahashi et al. 2017). However, other studies have not found a statistically significant association (Sun et al. 2019).

#### 3.7.3 Tumor Size

In all analyzed cases, a significant correlation (*p* < 0.05) between CAF concentration (measured as α‐SMA expression) and tumor size was found (Li et al. 2015; Akrish et al. 2017; Takahashi et al. 2017).

#### 3.7.4 Tumor Stage

The data regarding tumor stage are consistent, showing a significant correlation (*p* < 0.05) between CAF concentration (measured as α‐SMA expression) and TNM classification (Ding et al. 2014; Li et al. 2015; Akrish et al. 2017; Takahashi et al. 2017; Sun et al. 2019).

#### 3.7.5 Invasion Capacity

Multiple clinical studies have identified that a high number of CAFs in the stroma is associated with increased capacity for invasion and migration of tumor cells, not only in OSCC but also in various other cancer types, including ovarian, colorectal, breast, and hepatocellular carcinoma. This indicates that CAFs influence disease progression (Vered et al. 2010; Bagordakis et al. 2016; Takahashi et al. 2017). Moderate α‐SMA expression (CAFs concentration index) has been linked to deeply invasive tumors and increased perineural invasion (Akrish et al. 2017). A proposed mechanism by which CAFs enhance cancer invasiveness is through miR‐382‐5p, as previously mentioned (Sun et al. 2019).

#### 3.7.6 Metastasis

Studies agree that increased CAF density in tumor tissue is associated with a higher frequency of lymphatic invasion and lymph node metastasis (Ding et al. 2014; Li et al. 2015; Akrish et al. 2017; Takahashi et al. 2017; Sun et al. 2019).

Specifically, metastatic lymph nodes contain CAFs similar to those in primary tumors, and EMT markers are common in both primary and metastatic OSCC cells. Previous studies have proposed (but not demonstrated) two possible pathways for CAFs to reach lymph nodes: either CAFs migrate to the metastatic site along with carcinoma cells, or microenvironmental changes in metastatic tumors (similar to primary tumors) induce the recruitment and trans‐differentiation of various cells (Vered et al. 2010).

#### 3.7.7 Mortality and Recurrence

Most studies analyzing the relationship between CAFs (αSMA+ cells) and survival report that CAF‐rich OSCCs are significantly associated with higher mortality (Bello et al. 2011; Dayan et al. 2012; Ding et al. 2014; Vered et al. 2019).

Additionally, some studies report a relationship between high density of αSMA+ CAFs and disease recurrence (Dayan et al. 2012; Li et al. 2015; Bagordakis et al. 2016; Okuyama et al. 2018; Vered et al. 2019; Sakai et al. 2022).

Besides density, the spatial distribution of CAFs in the TME was found to be significantly different between cases with local recurrence and those without. Specifically, a high presence of CAFs at the invasive front was a negative prognostic factor (Bagordakis et al. 2016; Okuyama et al. 2018).

Given these data, some authors recommend routinely examining CAF density in OSCC cases to plan treatment and better define prognosis.

Conversely, other studies have not observed a statistically significant association between CAF concentration and survival rate (Akrish et al. 2017).

Of note, overall survival is also significantly influenced by patient age (Takahashi et al. 2017).

### 3.8 CAFs in OPMDs

Few studies have investigated the presence of CAFs in OMPDs. These studies primarily compared CAF concentrations in the microenvironment of selected OPMDs with those in the TME of OSCC. Of note, they focused on PVL and severe OED, which are recognized as having the highest risk for malignant transformation.

The data indicate that the number and distribution of CAFs (identified as αSMA+ cells) across different lesion types are statistically significant. CAFs are nearly undetectable in normal oral mucosa but increase progressively from OED to verrucous carcinoma and invasive OSCC (Kapse et al. 2013).

A different TME and growth behavior have been reported between OSCCs developed in patients with a history of PVL (p‐SCCA) and those with conventional squamous cell carcinoma (c‐SCCA) of the buccal mucosa, gingiva, and palate. Specifically, α‐SMA is significantly less expressed in p‐SCCA compared to c‐SCCA. Furthermore, p‐SCCA has been observed to exhibit a seemingly slower‐growing and less invasive pattern, which may be related to the lower concentration of CAFs (Akrish et al. 2017).

In conclusion, current evidence suggests that the number and distribution of CAFs could be theoretically useful in assessing OPMDs and OSCC throughout the process of oral carcinogenesis.

## 4 Discussion

Our scoping review consolidates knowledge on the critical role of CAFs in the OSCC TME and explores their potential involvement in the malignant transformation of OPMDs.

Literature shows that CAFs are crucial for OSCC development and progression through various mechanisms, including the secretion of pro‐tumorigenic molecules and direct interactions with cancer cells.

The correlation between high levels of CAFs and poor clinical outcomes in OSCC underscores the potential of CAFs as prognostic biomarkers, a possibility already proposed by some researchers. This also suggests the potential for targeting CAFs as a therapeutic strategy.

Similarly, evaluating CAFs could be innovative for assessing OPMDs. While data on OPMD microenvironments are limited and focus on specific OPMDs, the findings are promising.

Currently, the standard clinical decision‐making process does not incorporate CAFs (or any other TME parameters) in therapeutic decisions or prognostic predictions. Further research is needed to fully understand the mechanisms by which CAFs influence the OSCC TME and, even more so, the microenvironment of OPMDs.

In the authors' opinion, this topic is worth the effort and could offer new perspectives in several ways. Integrating CAF evaluation could implement current OSCC decision‐making guidelines by introducing a new parameter, and could provide objective diagnostic criteria for OPMDs assessment, potentially enabling more personalized management and offering a more effective means of predicting malignant transformation.

To achieve this, current knowledge about the role of CAFs in the TME of both OSCC and OPMDs should be expanded.

### 4.1 Limitations

The first limitation, as mentioned in the text, is that due to the increasing interest in the topic discussed in this scoping review and the regular publication of new papers, some studies may have been missed.

Second, the complexity of the topic makes it impossible to provide a complete and in‐depth analysis of all aspects within the limited space available, although we have endeavored to cover the main points.

## 5 Conclusion

Current evidence strongly supports the crucial role of CAFs in OSCC pathophysiology, yet significant challenges and limitations persist.

The heterogeneity of CAF populations, variability in their markers, and the complexity of their interactions with other TME components hinder our comprehensive understanding of their exact role in cancer progression. Additionally, the non‐univocal methods for CAF identification and characterization limits the comparability of studies.

Future research should aim to elucidate the specific mechanisms by which CAFs contribute to OSCC progression and investigate the potential of targeting CAFs for therapeutic purposes. Moreover, further studies are needed to clarify the role of CAFs in OPMDs and to assess whether and how their evaluation could improve patient management and OPMD outcomes.

## Author Contributions

**Samuele Sutera:** conceptualization, methodology, data curation, investigation, writing – original draft, project administration, formal analysis, validation, visualization, resources. **Olga Anna Furchì:** writing – review and editing, validation. **Monica Pentenero:** conceptualization, methodology, data curation, investigation, writing – review and editing, supervision.

## Ethics Statement

The authors have nothing to report.

## Consent

The authors have nothing to report.

## Conflicts of Interest

The authors declare no conflicts of interest.

## Data Availability

Research data not shared.
